# Subtotal tumor resection as a predictor of post-resection hydrocephalus in pediatric patients with posterior fossa tumors

**DOI:** 10.1007/s00381-025-06991-2

**Published:** 2025-10-25

**Authors:** Barnabas Obeng-Gyasi, Trenton A. Line, Anoop S. Chinthala, Jignesh Tailor

**Affiliations:** 1 Department of Neurological Surgery, Indiana University School of Medicine, Indiana University Health Neuroscience Center, 355 W 15 St, Suite 5100, Indianapolis, IN 46202 USA; 2 Division of Pediatric Neurosurgery, Riley Hospital for Children, Indianapolis, IN USA; 3 Herman B Wells Center for Pediatric Research, Indiana University School of Medicine, IN Indianapolis, USA; 4 Melvin and Bren Simon Comprehensive Cancer Center, Indiana University, IN Indianapolis, USA

**Keywords:** Pediatric brain tumor, Posterior fossa tumor, Hydrocephalus, Subtotal resection, Risk prediction, CSF diversion, Surgical outcomes

## Abstract

**Objective:**

Pediatric posterior fossa tumor (PFT) resection is frequently complicated by postoperative hydrocephalus. Previous hydrocephalus risk stratification tools demonstrate limited performance in external validation and do not account for important intra- and post-operative variables. We aimed to evaluate additional risk factors for post-resection hydrocephalus to improve risk-stratification.

**Methods:**

We conducted a retrospective analysis of pediatric patients who underwent resection of primary PFT’s at our institution (January 2016–June 2024). We collected perioperative variables thought to influence hydrocephalus risk. The primary outcome was permanent CSF diversion within 6 months after resection. We used univariable and multivariable logistic regression with bidirectional stepwise selection to identify predictors of post-resection hydrocephalus.

**Results:**

A total of 112 patients were included. We identified age < 5 years, moderate/severe hydrocephalus, and subtotal resection (STR; ≥ 1.5 cm^2^ residual tumor) as independent predictors of post-resection hydrocephalus on multivariable regression. STR exhibited an odds ratio of 8.25 (95% CI 2.72–26.84; *p* < 0.001), highlighting its strong association with the need for permanent CSF diversion. A scoring tool that incorporated STR and an age cutoff of < 5 years improved the discriminative performance (area under the ROC curve = 0.826) compared to the modified Canadian Preoperative Prediction Rule for Hydrocephalus (AUC = 0.720).

**Conclusions:**

These findings suggest STR is associated with increased risk of persistent hydrocephalus and emphasize careful intraoperative decision-making when pursuing incomplete resection. Future multicenter studies will be necessary to validate this framework and to further elucidate how STR, in conjunction with age and hydrocephalus severity, can be leveraged to optimize treatment strategies in diverse healthcare settings.

## Introduction

Pediatric posterior fossa tumors represent a significant cause of morbidity and mortality worldwide. Postoperative hydrocephalus is a common and potentially life-threatening complication, often requiring permanent cerebrospinal fluid (CSF) diversion [1, 2]. Accurate identification of patients at risk for permanent CSF diversion can inform surgical planning, expedite neuro-oncological treatment, reduce unnecessary procedures, and potentially improve overall patient outcomes [3]. The Canadian Preoperative Prediction Rule for Hydrocephalus (CPPRH) and its subsequent revision, the modified Canadian Preoperative Prediction Rule for Hydrocephalus (mCPPRH), were developed to aid in predicting the likelihood of post-resection hydrocephalus in these patients [2, 4]. Although external validation studies in multiple countries have reported variable discriminative accuracy (AUC ranging from ~0.62 to 0.86), these tools remain valuable in ruling out permanent CSF diversion in low-risk cohorts [5–7].

Despite the utility of the mCPPRH, there is increasing recognition that it may not fully capture certain clinical nuances that influence postoperative hydrocephalus by only focusing on preoperative characteristics [6, 7]. One emerging consideration is the impact of subtotal resection (STR), particularly as the pediatric neurosurgical field moves toward minimally invasive techniques, novel neoadjuvant therapies emerge after debulking surgery, and resource constraints differ across global regions [8, 9]. In many high-income settings, extensive resection is prioritized whenever feasible to reduce tumor burden, but an increasing emphasis on minimizing morbidity can lead to deliberate or unavoidable STR [10, 11]. Conversely, in areas with limited resources, the confluence of late diagnoses, suboptimal imaging, gaps in neurosurgical expertise, and infrastructural barriers may also increase the prevalence of STR [8, 12, 13].

Previous studies have not universally accounted for the role of STR as a risk factor for postoperative CSF diversion, nor for the potential interplay between STR, the need for perioperative EVD, and institutional practices that might influence shunt placement rates [2, 4, 6]. Other studies have examined the relationship between extent of resection (EOR) and risk for postoperative CSF diversion, but the definitions and categorization of EOR have been inconsistent, yielding mixed results [5, 14–18].

This study aimed to determine whether STR is an independent risk factor for the requirement of permanent CSF diversion within 6 months of surgery in pediatric patients with PFTs. Furthermore, we evaluated how integrating STR data into an adjusted mCPPRH model might refine its predictive accuracy. We also contextualized these findings in a global perspective, discussing the implications for resource-limited settings, where neurosurgical interventions such as shunt placement or advanced endoscopic procedures may not be uniformly available [19]. By clarifying the role of STR and identifying possible refinements to risk-prediction tools, we strive to inform future protocols that reduce morbidity and improve the quality of life for pediatric patients with posterior fossa tumors.

## Methods

### Study design and patient population

We conducted a retrospective analysis of pediatric patients who underwent resection of posterior fossa tumors at Riley Hospital for Children in Indianapolis, Indiana, between January 2016 and June 2024. Patients were identified through a search of the electronic medical records. Inclusion criteria were age ≤ 18 years at diagnosis and radiographically confirmed posterior fossa tumor. Exclusion criteria included prior permanent CSF diversion procedures [endoscopic third ventriculostomy (ETV) or ventricular shunt], permanent CSF diversion during initial resection, recurrent tumor or prior tumor resection, biopsy of tumor without resection, and incomplete medical records. Patients who underwent resection within 6 months of data collection and had not yet required permanent CSF diversion, or who died before 6 months, were excluded because they had not yet progressed through the time frame of the primary outcome.

### Data collection

Degree of tumor resection was collected from postoperative MRI with contrast as described and measured in the radiology reports. All postoperative imaging reports without clear determinations of residual tumor had follow-up imaging that confirmed or ruled out the presence of residual tumor. Subtotal tumor resection was defined as residual tumor ≥ 1.5 cm^2^ primary tumor residuum, near total resection was defined as < 1.5cm^2^ primary tumor residuum, and gross total resection was defined as no evidence of residual tumor. Degree of hydrocephalus, presence of metastatic disease, presence of transependymal edema, and tumor location were determined by the radiologist’s assessment as commented in the preoperative imaging reports. Qualitative assessment of the degree of hydrocephalus was dichotomized into groups of moderate/severe and none/mild, following the methods of a previous validation study [7]. Moderate/severe hydrocephalus was defined by descriptions of moderate, severe, or significant hydrocephalus. Descriptions of mild, minimal, small, minor, or absent hydrocephalus were categorized as none/mild. All radiologist reports contained assessment of the degree of hydrocephalus. Metastatic disease was defined by the radiologist’s report of leptomeningeal enhancement, drop metastases, or other tumor foci beyond the primary site of the tumor, including intracranial and intraspinal locations. Transependymal edema was defined as periventricular hyperintensity on T2 FLAIR MRI sequences. Midline tumor location was defined as the primary tumor centered in or significantly involving the fourth ventricle, floor or roof of the fourth ventricle, pineal body, cerebellar vermis or nodule, or midline brainstem. Tumor diagnosis, WHO tumor grade, and molecular subtype of medulloblastoma were collected from pathology reports. Hemorrhagic tumor was collected from preoperative MRI and was defined as the presence of tumoral hemorrhage; tumors with only microhemorrhages or small punctate hemorrhages were not counted as hemorrhagic. Postoperative intraventricular blood was collected from postoperative imaging, defined as ≥ 1 mm of blood in the ventricles. The primary outcome was the need for permanent CSF diversion, defined as the placement of a ventricular shunt or endoscopic third ventriculostomy within 6 months of initial tumor resection. Permanent CSF diversion was performed according to physician judgment based on clinical symptoms and radiological evidence of hydrocephalus in the postoperative period, or after a failed EVD clamp trial in those with an EVD in place.

### Statistical analysis

Patient characteristics were grouped based on the primary outcome and compared using Fisher’s exact test and chi-squared test for categorical variables and Wilcoxon rank-sum test for continuous variables. Age was dichotomized using ROC curve and Youden’s index to identify the ideal cutoff. Variables with differences between groups with *p* < 0.3 were then analyzed using univariable logistic regression. Univariable odds ratios (OR) and *p*-values were calculated for each predictor. Variables with univariable *p*-values < 0.3 were included in a multivariable logistic model, and bidirectional stepwise selection was performed to select model features.

Several prediction tools were created using the important predictors from the logistic regression model, combined with predictors from the mCPPRH, assigning points to each predictor for a score out of 10. ROC curve analysis was done to calculate AUC for each tool. The sensitivity, specificity, positive predictive value, negative predictive value, and positive and negative likelihood ratios were calculated at different cutoff scores for comparison to the mCPPRH. The mCPPRH score was calculated for each patient based on the following factors: presence of moderate/severe hydrocephalus (2 points), presence of metastatic disease (3 points), age < 2 years (3 points), presence of transependymal edema (1 point), diagnosis of medulloblastoma (1 point), diagnosis of ependymoma (1 point), and diagnosis of dorsal exophytic brainstem glioma (1 point). To calculate the estimated risk of CSF diversion for each total score, logistic regression using score components as parameters calculated the log odds of outcome at each score strata, which were transformed into probabilities using the formula $P =\frac{1}{1 + {e}^{ -\left(\mathrm{log}odds\right)}}$. All analyses were performed using RStudio 2024.09.0 + 375 (RStudio, PBC).

## Results

### Patient selection and demographics

A total of 203 pediatric patients who underwent resection of posterior fossa tumors were initially identified through electronic medical records at our institution (January 2016–June 2024). After applying the inclusion and exclusion criteria, 112 patients (age ≤ 18 years) with radiographically confirmed posterior fossa tumors remained. Of the 91 excluded, 60 did not have true posterior fossa tumors, 13 did not undergo surgical resection, 3 had prior permanent CSF diversion, 11 had recurrent tumors, and 4 either had resections or died within 6 months of data collection. Patient demographics and cohort characteristics are summarized in Table 1. Overall, 31.3% of patients (*n* = 35) required permanent CSF diversion within 6 months of surgery. ETV was performed on one patient, while the other 34 received shunts.

**Table 1:** Patient demographics and clinical characteristics of cohort, stratified by the need for permanent CSF diversion within 6 months of resection. The count and percent of outcome strata are shown for categorical predictors, and the median and interquartile range (IQR) are shown for age, admission GCS, and intraoperative blood loss (mL)

	**Permanent CSF diversion within 6 months**		
**Characteristic**	**No**^**1**^** (*n* = 77)**	**Yes**^**1**^** (*n* = 35)**	***p-*value**^**2**^
Sex (F)	35 (45%)	13 (37%)	0.40
Age (years)	8.0 (5.0, 12.0)	4.0 (2.0, 7.0)	< 0.001***
Age < 5 years	19 (25%)	22 (63%)	< 0.001***
**Admission GCS**			0.50
Median (Q1, Q2)	15 (15, 15)	15 (15, 15)	
Min, max	6, 15	3, 15	
**Tumor diagnosis**			> 0.90
Astrocytoma	31 (40%)	14 (40%)	
Medulloblastoma	19 (25%)	10 (29%)	
Ependymoma	12 (16%)	5 (14%)	
AT/RT	4 (5.2%)	2 (5.7%)	
Brainstem glioma	2 (2.6%)	0	
Diffuse glioma	1 (1.3%)	0	
Hemangioblastoma	3 (3.9%)	1 (2.9%)	
Teratoma	0	1 (2.9%)	
Other	5 (6.5%)	2 (5.7%)	
**WHO grade**			0.50
1	36 (49%)	15 (45%)	
2	8 (11%)	1 (3.0%)	
3	5 (6.8%)	4 (12%)	
4	25 (34%)	13 (39%)	
n/a	1	2	
**Medulloblastoma molecular subtype (*n* = 29)**			0.80
WNT	2 (11%)	0	
SHH	10 (53%)	7 (70%)	
Non-WNT, non-SHH	4 (21%)	2 (20%)	
Group 3	1 (5.3%)	1 (10%)	
Group 4	2 (11%)	0	
Moderate/severe hydrocephalus (Y)	54 (70%)	33 (94%)	0.004**
Transependymal edema (Y)	50 (65%)	31 (89%)	0.01*
Metastases (Y)	4 (5.2%)	7 (20%)	0.03*
Perioperative EVD (Y)	59 (77%)	33 (94%)	0.02*
Hemorrhagic tumor (Y)	17 (22%)	4 (11%)	0.20
Midline tumor location (Y)	56 (73%)	29 (83%)	0.20
Postoperative intraventricular blood (Y)	39 (51%)	19 (54%)	0.80
Intraoperative blood loss (mL)	100 (50, 200)	100 (50, 200)	> 0.9
**Extent of resection**			< 0.001***
Gross total resection	57 (74%)	14 (40%)	
Near total resection	11 (14%)	3 (8.6%)	
Subtotal resection	9 (12%)	18 (51%)	

^1^*n* (% of outcome strata), median (IQR)

^2^*p < 0.05; **p < 0.01; ***p < 0.001

F female, GCS Glasgow Coma Scale, AT/RT atypical teratoid rhabdoid tumor, WHO World Health Organization, Y yes, EVD external ventricular drain

### Univariable analysis of risk factors

We tested potential predictors of the primary outcome, permanent CSF diversion within 6 months, using univariable logistic regression (Table 2). Subtotal resection (OR 8.14; CI (3.11–22.88); *p* < 0.001) emerged as the strongest predictor among all tested variables, while near total resection did not reach statistical significance. Age (OR 0.86; CI (0.77–0.93); *p* = 0.0014), particularly age < 5 (OR 5.17; CI (2.22–12.51); *p* < 0.001), was strongly associated with increased risk of hydrocephalus. Other factors that reached statistical significance (*p* < 0.05) included moderate/severe hydrocephalus on preoperative imaging (OR: 7.03; CI (1.91–45.58); *p* = 0.011), transependymal edema (OR 4.19; CI (1.46–15.16); *p* = 0.014), metastases (OR 4.56; CI (1.28–18.56); *p* = 0.023), and perioperative EVD placement (OR 5.03; CI (1.34–32.91); *p* = 0.037).

**Table 2:** Univariable logistic regression results for individual predictors. Odds ratios (OR), 95% confidence intervals (CI), and *p*-values are shown for each predictor

Univariable logistic regression			
**Predictor**	**OR**	**CI**	***p*-value**^**1**^
Age	0.86	0.77–0.93	0.001**
Age < 5 years	5.17	2.22–12.51	< 0.001***
**Extent of resection**			
Subtotal	8.14	3.11–22.88	< 0.001***
Near total	1.11	0.23–4.15	0.88
Moderate/severe hydrocephalus	7.03	1.91–45.58	0.01*
Transependymal edema	4.19	1.46–15.16	0.01*
Metastases	4.56	1.28–18.56	0.02*
Perioperative EVD	5.03	1.34–32.91	0.04*
**Tumor diagnosis**			
Medulloblastoma	1.17	0.43–3.14	0.90
Ependymoma	0.92	0.25–3.03	0.76
Hemorrhagic tumor	0.46	0.12–1.36	0.18
Midline tumor location	1.81	0.69–5.38	0.25

^1^*p < 0.05; **p < 0.01; ***p < 0.001

GCS Glasgow Coma Scale, EVD external ventricular drain

### Multivariable logistic regression model

**Fig. 1:**
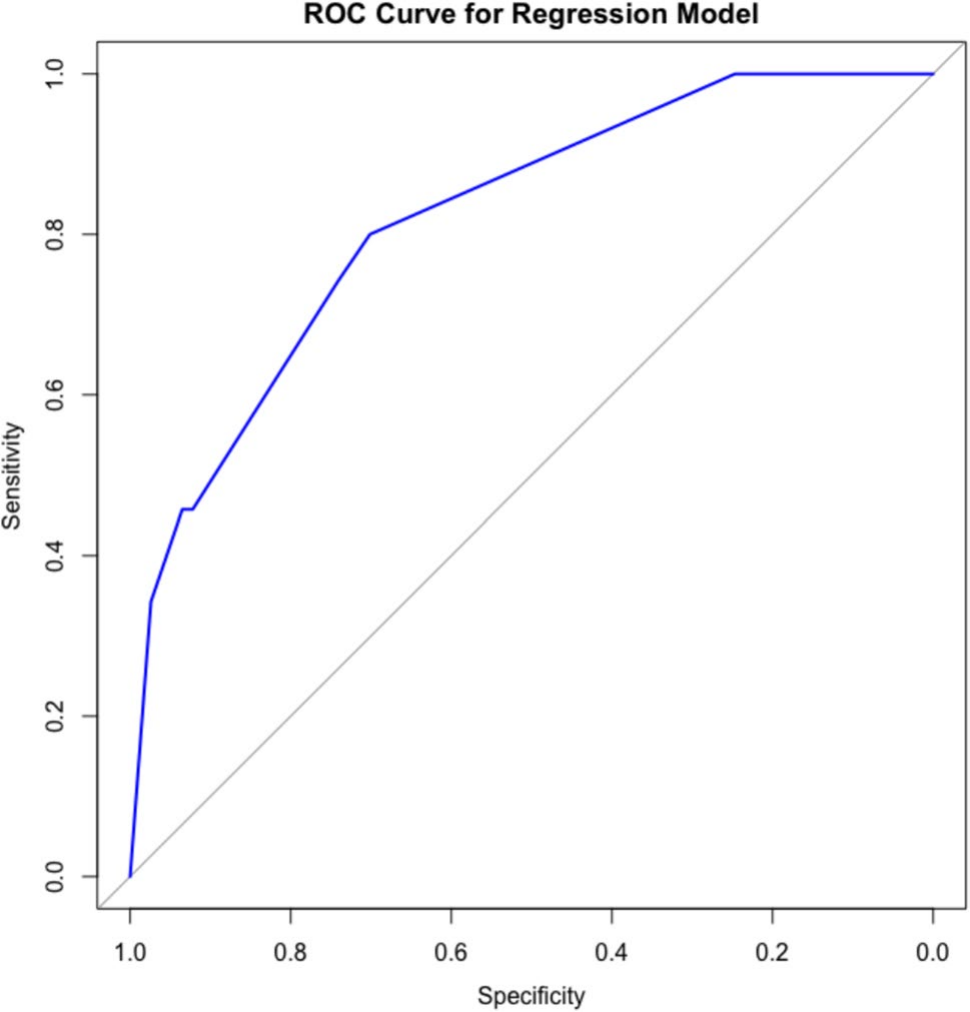
ROC curve for the final multivariable logistic regression model. The figure displays the reciever operating characteristics (ROC) curve for the logistic regression model with parameters age < 5 years, subtotal resection, and moderate/severe hydrocephalus was used to predict the need for permanent CSF diversion. Predicted probabilities were compared against the observed outcome. Area under the ROC curve (AUC) was 0.821. The specificity decreasing from 1.0 to 0.0 is plotted along the *X*-axis and the sensitivity from 0.0 to 1.0 is plotted along the *Y*-axis

Age < 5 years, subtotal resection, moderate/severe hydrocephalus, transependymal edema, metastases, perioperative EVD, hemorrhagic tumor, and midline tumor location were entered into a multivariable logistic regression model. A variance inflation factor test confirmed no significant multicollinearity among model parameters. Stepwise selection identified three independent predictors of permanent CSF diversion: (1) age < 5 y/o (aOR 3.20; CI (1.16–8.82); *p* = 0.020), (2) subtotal resection (aOR 8.25; CI (2.72–26.84); *p* < 0.001), and (3) moderate/severe hydrocephalus (aOR 6.84; CI (1.95–29.61); *p* = 0.020) (Table 3). The area under the ROC curve (AUC) for the final multivariable model was 0.821, indicating excellent discriminative ability in predicting the need for permanent CSF diversion (Fig. 1).

**Table 3:** Results of final multivariable logistic regression model after bidirectional stepwise selection. Variables included in multivariable model if *p* < 0.3 in univariable regression

Stepwise multivariable logistic regression			
**Predictor**	**aOR**	**95% CI**	***p*-value**^**1**^
Age < 5 years	3.20	1.16–8.82	0.02*
Subtotal resection	8.25	2.72–26.84	< 0.001***
Moderate/severe hydrocephalus	6.84	1.95–29.61	0.02*

^1^*p < 0.05; **p < 0.01; ***p < 0.001

### Comparison of scoring tools

**Fig. 2:**
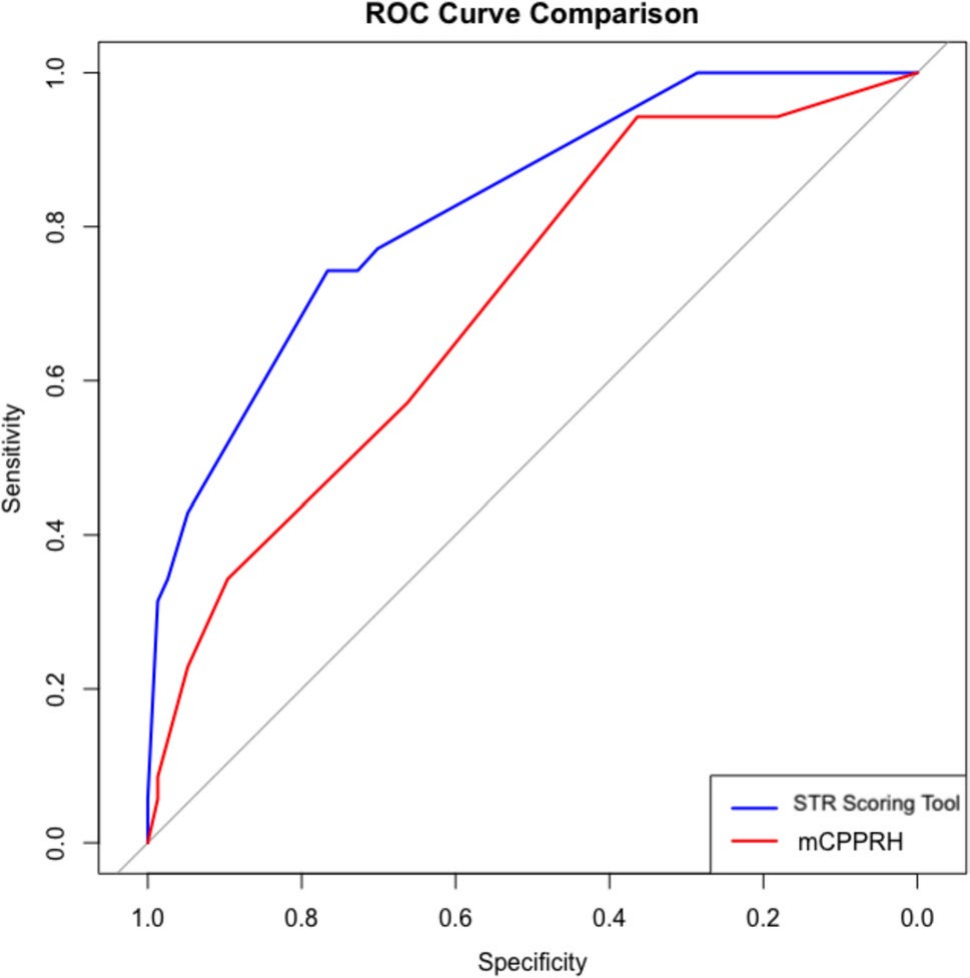
ROC curve comparison for scoring tools. The figure displays the receiver operating characteristics (ROC) curve for our STR scoring tool and the mCPPRH. The STR scoring tool assigned points for age < 5 (3 points), subtotal resection (3 points), moderate/severe hydrocephalus (2 points), metastases (1 point), and transependymal edema (1 point). The mCPPRH assigns points for age < 2 (3 points), diagnosis of ependymoma or medulloblastoma (1 point), moderate/severe hydrocephalus (2 points), metastases (3 points), and transependymal edema (1 point). The AUC for the STR scoring tool was 0.826, compared to 0.720 for the mCPPRH. The specificity decreasing from 1.0 to 0.0 is plotted along the *X*-axis, and the sensitivity from 0.0 to 1.0 is plotted along the *Y*-axis

We then developed a new scoring tool by modifying the components of the mCPPRH to incorporate these independent predictors (Table 4). Our STR scoring tool, which replaces tumor diagnosis with STR, assigns 3 points for STR, 3 points for age < 5 years (rather than < 2 years), and 1 point for metastases (instead of 3 points), yielded the highest AUC (0.826), outperforming the standard mCPPRH (AUC 0.720). A ROC curve comparison of the STR scoring tool vs. the mCPPRH is shown in Fig. 2, illustrating the improved overall model performance. We further computed the predicted probability of permanent CSF diversion across a range of total scores (0–10) using the STR scoring tool, demonstrating how incremental differences in the score translate to rising or falling hydrocephalus risk (Table 5).

**Table 4:** Comparison of the STR scoring tool to the modified preoperative prediction tool for hydrocephalus (mCPPRH). Model components and their corresponding point values are shown in the first two columns

**STR scoring tool**									
Predictors	Points	AUC	Cutoff	SENS	SPEC	PPV	NPV	+ LR	−LR
Age < 5 years	3	0.826	4	77.1	70.1	54.0	87.1	2.58	0.32
Subtotal resection	3		5	74.3	72.7	55.3	86.2	2.72	0.35
Moderate/severe hydrocephalus	2		6	74.3	76.6	59.1	86.8	3.18	0.34
Metastases	1		7	42.9	94.8	78.9	78.5	8.25	0.60
Transependymal edema	1		8	34.3	97.4	85.7	76.5	13.2	0.67
**Modified Canadian Preoperative Prediction Rule for Hydrocephalus (mCPPRH)**									
Predictors	Points	AUC	Cutoff	SENS	SPEC	PPV	NPV	+ LR	−LR
Age < 2 years	3	0.720	4	57.1	66.2	43.4	77.3	1.69	0.64
Ependymoma/medulloblastoma	1		5	34.3	89.6	60.0	75.0	3.30	0.73
Moderate/severe hydrocephalus	2		6	28.6	92.2	62.5	74.0	3.67	0.77
Metastases	3		7	22.9	94.8	66.7	73.0	4.40	0.81
Transependymal edema	1		8	8.6	98.7	75.0	70.4	6.6	0.92

AUC area under the ROC curve, SENS sensitivity, SPEC specificity, PPV positive predictive value, NPV negative predictive value, + LR positive likelihood ratio, − LR negative likelihood ratio

**Table 5:** Predicted risk for permanent CSF diversion at each score strata using the STR scoring tool

Total score	Estimated risk
0	0.018
1	0.048
2	0.121
3	0.273
4	0.505
5	0.735
6	0.883
7	0.953
8	0.982
9	0.993
10	0.998

## Discussion

Our findings indicate that subtotal resection (STR) is significantly associated with the need for permanent CSF diversion following PFT resection in children, a result that complements and extends the existing literature on hydrocephalus risk factors [14, 16, 17]. In a cohort where 31.3% ultimately required permanent CSF diversion, STR emerged as an independent predictor after adjusting for known factors such as moderate-severe hydrocephalus on preoperative imaging and metastatic disease. These results underscore the importance of considering tumor resection status in perioperative planning and prognostication tools like the mCPPRH.

### Subtotal resection as a predictor of postoperative CSF diversion

The relationship between the extent of resection and the risk of postoperative hydrocephalus has not been well established. In our cohort, STR was independently associated with the need for permanent CSF diversion on multivariable analysis (OR 8.25; CI (2.72–26.84); *p* < 0.001). Near total resection (NTR), however, was not associated with the primary outcome on univariable regression. Several studies have found the extent of resection (EOR) to be predictive of permanent CSF diversion following PFT surgery in children, but EOR has been inconsistently defined.

Guo et al. found gross total resection to be negatively associated with postoperative hydrocephalus (OR 0.284; CI (0.095–0.855); *p* = 0.025), but they did not distinguish between STR and NTR [16]. Darshan et al. did not find any association between EOR and shunt placement (OR 0.948; CI (0.227–3.95); *p* = 0.941) but did not clearly define NTR or STR [5]. Kumar et al. found a significant correlation between partial resection (including biopsy, debulking, subtotal, and near-total excisions) and postoperative shunt placement [17]. Chen et al. reported partial resection was independently associated with persistent hydrocephalus, while STR was not, although these variables were not clearly defined [14].

In contrast to our results, two studies reported no association between incomplete resection and postoperative CSF diversion, but they did not analyze STR independently from NTR [15, 18]. Given that NTR was not associated with the primary outcome in our cohort, previous studies may have obfuscated the impact of EOR by failing to distinguish between STR and NTR. Future studies should clearly define EOR and should attempt to analyze STR and NTR independently.

### Implications for clinical practice

The implications of these findings extend to several aspects of clinical practice. First, they highlight the potential need for preoperative planning that considers the trade-off between the extent of resection and hydrocephalus risk. STR may be pursued for various reasons, including minimizing neurological deficits, avoiding damage to critical brainstem structures, or achieving rapid symptom relief in unstable patients [10, 20, 21]. The rationale often stems from legitimate concerns about postoperative quality of life when aggressive resection might risk permanent neurological impairment. While GTR is generally the goal of surgery, it may be possible to achieve similar outcomes with less aggressive resection thanks to advances in tumor biology and neoadjuvant therapies [22]. However, our findings suggest that the decision to prioritize immediate neurological preservation should be carefully weighed against the increased risk of persistent hydrocephalus.

Second, institutions may need to reassess their protocols for EVD management in cases of STR, potentially implementing more structured weaning trials or earlier evaluation for permanent CSF diversion. While EVDs provide crucial management of acute hydrocephalus and allow for careful intracranial pressure monitoring, they may inadvertently mask the development of permanent CSF circulation disorders [23, 24]. This temporary solution might delay recognition of which patients will ultimately require permanent CSF diversion or may increase the risk of persistent hydrocephalus by introducing additional tissue damage, hemorrhage, and inflammation [5, 6].

Third, our findings raise important questions about the role of staged surgical approaches. In cases where initial imaging suggests high-risk features for incomplete resection, a planned staged approach might provide a better balance between immediate safety and long-term CSF dynamics than a single STR procedure. This strategy, while potentially requiring multiple operations, could help identify patients who might benefit from early permanent CSF diversion versus those in whom careful observation is appropriate.

Furthermore, these findings emphasize the importance of transparent discussion with families about the potential long-term consequences of surgical approach selection. While the immediate benefits of planned STR may be apparent, families should understand that these decisions might influence the likelihood of requiring permanent CSF diversion—a consideration that could affect their preferences regarding surgical strategy.

### Integration of STR into risk prediction models

The traditional mCPPRH incorporates several well-established risk factors, including age below 2 years, specific tumor diagnoses such as medulloblastoma and ependymoma, the presence of metastatic disease, and markers of elevated intracranial pressure. By focusing on preoperative characteristics and ignoring important intra-operative and postoperative variables, such as EOR, the mCPPRH may not adequately capture hydrocephalus risk, limiting its utility [2, 4–7]. The mCPPRH was originally developed to inform decisions of whether to perform permanent CSF diversion alongside primary resection. In practice, however, the tool is often utilized postoperatively to guide CSF diversion decisions and counsel patients and families. Identifying high-risk patients in the postoperative period can guide surgeons in performing CSF diversion procedures while patients are still in the hospital receiving additional therapies, which could expedite the treatment course and prevent additional hospital admissions.

Our modified scoring system, which incorporates STR as a key variable, demonstrated improved predictive accuracy compared to the standard mCPPRH (AUC 0.826 vs. 0.720). This enhancement in predictive capability holds particular relevance for real-world clinical scenarios, where complete resection may be precluded by anatomical constraints or safety considerations. The refined scoring system may better reflect the nuanced clinical decision-making inherent in treating posterior fossa tumors.

### Global resource implications and care delivery

The practice of pediatric neurosurgery exists within markedly different resource environments worldwide, creating distinct challenges in the management of posterior fossa tumors. In high-resource settings, care typically includes advanced imaging modalities, multidisciplinary tumor boards, and sophisticated surgical equipment including neuronavigation systems and surgical microscopes. These technological advantages, combined with the advanced experience of neurosurgeons, specialized pediatric intensive care units, and dedicated neuroanesthesia teams, often enable the pursuit of gross total resection when anatomically feasible [12, 13, 25].

In contrast, low- and middle-income countries (LMICs) face numerous systemic barriers that significantly impact patient care. Late presentation of pediatric brain tumors remains a persistent challenge, driven by limited access to primary care services, geographical obstacles in reaching specialized treatment centers, and cultural factors that may delay initial medical evaluation [8, 26–29]. The diagnostic capabilities in these settings are often compromised by outdated imaging equipment or the absence of advanced imaging modalities, limiting surgeons’ abilities to accurately delineate tumor margins. These challenges are further exacerbated by shortages of trained pediatric neurosurgeons, specialized nursing staff, and essential surgical equipment [26–29]. Consequently, these constraints frequently result in higher rates of subtotal resection [8, 12, 13], which our data suggest significantly elevates the risk of persistent or progressive hydrocephalus.

The management of postoperative hydrocephalus presents additional complexities in resource-limited environments. While ventriculoperitoneal shunts remain a primary treatment option, their use carries substantial risks of infection and hardware malfunction, requiring ongoing maintenance and consistent follow-up care that may be difficult to provide in these settings [19, 30]. Endoscopic third ventriculostomy is often unavailable or underutilized due to equipment limitations and insufficient specialized training [19, 31]. This situation creates particular vulnerability for patients who undergo subtotal resection, as they may face significant barriers to accessing necessary salvage procedures for hydrocephalus.

### Study limitations and future directions

The retrospective nature of our analysis and single-center design inevitably poses limitations in generalizability. Our results are limited to the strategies employed at this single center. Variations in institutional protocols for CSF diversion, surgical techniques, thresholds for resection extent, and postoperative protocols may produce different results. Furthermore, the classification of the extent of resection relied partly on postoperative imaging and operative reports, which can be subjective. Larger, prospective multicenter trials that systematically record surgical intent, imaging-based resection percentages, and standardized definitions of hydrocephalus severity would provide more robust evidence. We plan to perform these validation studies in collaboration with other centers in the USA and internationally. Finally, it remains uncertain whether augmenting the mCPPRH to incorporate STR generalizes well to all pediatric PFT populations, particularly given the wide variability in global resource settings.

## Conclusions

Our results provide novel insight into the role of subtotal resection as an important risk factor for permanent CSF diversion in pediatric PFT surgery. Integrating STR into risk prediction models such as the mCPPRH could refine clinical decision-making, reduce morbidity, and ultimately improve outcomes in this vulnerable population. Future research should focus on validating these findings in diverse patient cohorts and exploring how evolving surgical practices influence the balance between oncologic efficacy and neurologic safety.

## Data Availability

No datasets were generated or analysed during the current study.
